# Leaf Spot Caused by *Alternaria* spp. Is a New Disease of Grapevine

**DOI:** 10.3390/plants13233335

**Published:** 2024-11-28

**Authors:** Evgeniya Yurchenko, Daria Karpova, Margarita Burovinskaya, Svetlana Vinogradova

**Affiliations:** 1 North Caucasian Federal Scientific Center of Horticulture, Viticulture, Wine-Making, 40 Years of Victory Street, Build. 39, 350901 Krasnodar, Russia; 2 Skryabin Institute of Bioengineering, Research Center of Biotechnology of the Russian Academy of Sciences, Leninsky Prospect, 33, Build. 2, 119071 Moscow, Russia

**Keywords:** *Vitis vinifera*, *Alternaria* leaf spot of grapevine, multilocus typing, *Alternaria alternata*, *Alternaria arborescens*, fungicides, *Trichoderma* sp.

## Abstract

In this study, we carried out large-scale leaf spot symptom observation on vineyards in the Krasnodar Krai of Russia and determined their distribution. The incidence and severity of leaf spot were higher on the Euro-American grapevine hybrids (Bianka, Levokumskij, Avgustin, Moldova, Pervenets Magaracha, Dunavski lazur). A total of 433 isolates that belonged to the genus *Alternaria* were isolated from samples with leaf spot. Pathogenicity testing confirmed the ability of the representative isolates to cause necrosis on the grapevine. The isolates of *Alternaria* sp. were typed by the loci of internal transcribed spacer (ITS), glyceraldehyde-3-phosphate dehydrogenase (*gapdh*), Alternaria allergen a1 (*Alt a1*), β-tubulin (*tub*), and translation elongation factor (*tef1*). Isolates from grapevine causing leaf spot were shown to cluster with isolates of *Alternaria. alternata* (Fr.) Keissl. and *Alternaria. arborescens* E.G. Simmons species complex. Of the fungicides tested to inhibit *Alternaria* growth, the most effective were mixtures, such as pyrimethanil and fluopyram, cyprodinil and fludioxonil, and those that included difenoconazole. The results of the study expand our knowledge of the biodiversity of *Alternaria* sp. fungi and can be used to limit the spread of *Alternaria* leaf spot of the grapevine.

## 1. Introduction

Grapevines are one of the oldest the oldest crops cultivated by humans; they play a significant role in the economy and culture of many countries. According to the International Organization of Vine and Wine, in 2023, the area of vineyards in Russia was 105 hectares, ranking 17th in the world [1]. Grapevines are susceptible to many pathogens, among which pathogens from kingdoms Fungi and Chromista cause the most harmful and economically significant diseases, such as mildew (*Erysiphe necator* Schwein.), downy mildew (*Plasmopara viticola* Berk. & M.A. Curtis), gray rot (*Botrytis cinerea* Pers.), black rot (*Guignardia bidwellii* (Ellis) Viala & Ravaz), and anthracnose (*Elsinoe ampelina* Shear) [2,3]. These diseases affect both the leaves and the generative organs of the grapevine.

Over time, the functional structure of fungal communities, including those in vineyards, accumulates changes associated with global climate change and the intensification of production; among them, the expansion of the species composition of fungi, including pathogenic ones, increases their aggressiveness and the appearance of new economically significant species [4,5].

Worldwide, there is a trend towards an increase in the harmfulness and range of phytopathogenic fungi [6,7], including the genus *Alternaria*, on various agricultural crops [8,9,10,11,12,13,14]. To date, more than 300 species assigned to the genus *Alternaria* are known [15]. Most species of this genus are considered cosmopolitan; they can occupy various ecological niches and play roles as saprotrophs, endophytes, and pathogens. It is often noted that pathogens act as necrotrophs, i.e., they feed on dead cells of the host plant [16]. *Alternaria* spp. affects all parts of a plant, including leaves, stems, and fruits; it is also well known as a pathogen that causes rot during the storage of the crop [17]. Common symptoms of diseases caused by *Alternaria* spp. are necrotic lesions on leaves in the form of large brown or black spots with characteristic concentric zonation, often surrounded by yellow chlorotic tissue. This zone arises as a result of the diffusion of fungal secondary metabolites that are toxic to the host [18,19,20].

Mycologists and plant pathologists disagree on the taxonomy of *Alternaria* spp. The classification based on the morphological characteristics of the conidia of the micromycetes and their substrate specialization was developed by Simmons as a result of many years of detailed studies on the sporulation pattern and conidial morphology of isolates [21]. However, owing to the highly variable morphological and cultural characteristics of fungi, which vary greatly depending on the substrate, illumination, and humidity, errors and uncertainties in taxonomy arise. In an attempt to overcome the difficulties of the taxonomic delimitation of *Alternaria* fungi, the use of pathological differences in isolates (the production of host-specific toxins (HTS)) and the application of the term “pathotype” were proposed [22,23]. Subsequent studies based on molecular phylogenetic approaches revealed that clades of *Alternaria* species do not always correlate with species groups defined on the basis of morphological characteristics [24,25]. A major step toward streamlining the taxonomy of the *Alternaria* complex was made in the studies of Lawrence et al. [26] and Woudenberg et al. [27], who defined internal clades in the *Alternaria* complex as sections. The use of multigene phylogeny and comparative genomics allowed the identification of *Alternaria* isolates in sections. For example, 11 phylogenetic species and one species complex were identified in an *Alternaria* section [28]. Moreover, 35 morphospecies were indistinguishable; therefore, it was proposed to synonymize them as *Alternaria alternata* (Fr.) Keissl., including such common phytopathogens of agricultural crops as *A. alternata*, *Alternaria tenuissima* (Kunze) Wiltshire, and *Alternaria citri* Ellis & N. Pierce.

For a more reliable identification of *Alternaria* fungi, molecular markers (RAPD, AFLP, ISSR, and SSR) are often used [29,30,31,32], as are approaches based on the amplification and sequencing of specific loci, such as internal transcribed spacer (ITS) [33], genes, or gene fragments encoding mitochondrial small subunit (mtSSU), translation elongation factor (*tef1-α*), β-tubulin (*tub*) [34], plasma membrane ATPase [35], calmodulin [36], endopolygalacturonase (*endo-PG*) [37], Alternaria allergen a1 (*Alt a1*) [38], RNA polymerase II largest subunit (RPB2), glyceraldehyde 3-phosphate dehydrogenase (gapdh) [39] and anonymous regions of the genome (ORA1-3 and OPA2-1) [40], large subunit ribosomal RNA (LSU), small subunit ribosomal RNA (SSU), etc. [41].

Due to the growing economic importance of *Alternaria* fungi for agricultural crop production, the search for effective control agents has intensified. The most effective agents are fungicides [42,43]. The efficacy of compounds with dicarboxyimide groups such as promicidone [44], benzimidazoles [45], and triazoles [46], is noted. Moreover, the use of biofungicides based on essential oils and biopolymers to combat phytopathogens has demonstrated a positive effect [47]. The use of antagonistic bacteria and fungi can be an alternative method for managing fungal diseases [48,49]. The control of fungal diseases requires an integrated approach that includes monitoring the pathogen, studying its biology and ecology, and using integrated protection methods designed to minimize their negative impact on the wine industry.

Earlier, we discovered in the Krasnodar Krai of Russia symptoms of leaf spot on grapevines [50]. These symptoms were associated with the presence of fungi of the genus *Alternaria* on the plant. In this work, the results of a large-scale leaf spot symptom observation of grapevines are presented for the first time, the causative agent of this disease is identified, and promising chemical preparations and biological agents for its control in the vineyard are determined.

## 2. Results

### 2.1. Symptoms and Samples Collection

**Figure 1:**
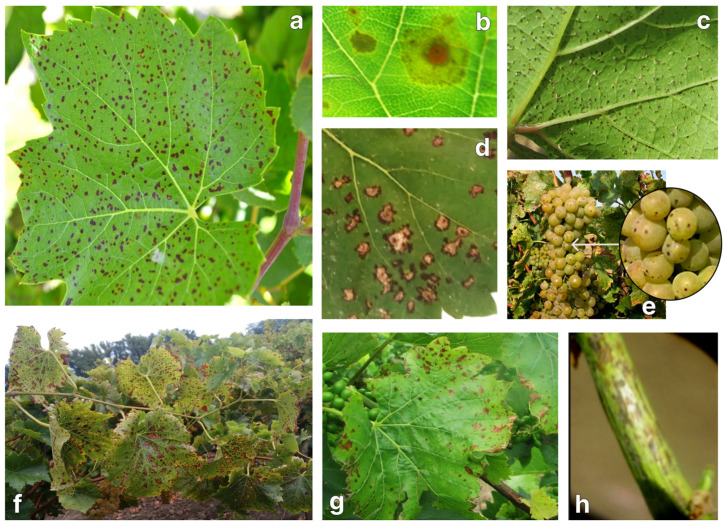
Symptoms of grapevine leaf spot: (**a**,**c**) leaf spot at the initial stage of infection; (**b**) necrotic spot on the leaf; (**d**) hypersensitivity reaction on the leaf; (**e**) grapevine bunch with necrotic spots; (**f**,**g**) leaf spot at the late stage of infection; and (**h**) grapevine stem with necrotic spots.

In June 2020, symptoms of leaf spot were detected in the BBCH68 growth stage on the varieties Bianka, Levokumskij, Pervenets Magaracha, Riton, Avgustin, Moldova, Alkor, and Dmitriy. Small rounded necroses of black or dark brown color were appearing first on the underside of the leaf, and then becoming visible on the upper side of the leaf, which indicates infection with conidia through the stomata (Figure 1a,c). Large spots had a black border, which is a manifestation of a hypersensitivity reaction (Figure 1d). This pattern of pathogenesis suggested the presence of a parasitic stage of the mycopathogen. At the late stages of infection, necrotic spots were merging, and the leaves were drying up and falling off. (Figure 1b,f,g). With the intensive development of the disease in individual foci in the second half of summer, damage to bunches and stems was observed (Figure 1e,h).

In 2021, the degree of the incidence and severity of leaf spot was determined (Table 1). The greatest severity of the disease was noted on Euro-American hybrids (Bianka, Levokumskij, Avgustin, Moldova, Pervenets Magaracha, and Dunavski Lazur) (Supplementary Figure S1a–c). The incidence of the disease on the Bianka and Levokumskij varieties reached almost 80–90%, whereas severity exceeded 61.9%. In contrast, Euro-Amur hybrids (Kunleany, Bruskam, Amur, Cristal, and Vostorg) were not affected at all or were affected only slightly (I 0–7.4, S 0–3.5%).

The incidence and severity of leaf spot on the *Vitis vinifera* species were higher on white varieties of the Western European eco-geographical group—convar. *occidentalis* subconvar. *gallica* (Sauvignon Blanc, Pinot Blanc, Chardonnay, Aligote, Muller Thurgau, and Risling Rejnski) (Supplementary Figure S1d–g). The incidence of the disease on these ranged from 0 to 29.1%, and the severity from 0 to 9.2%. The maximum incidence and severity of leaf spot were recorded for the Muller Thurgau (I 29.1% and S 9.2%) and Sauvignon Blanc (I 26.4% and S 9.0%) varieties. The first signs of disease on these were detected in the growth stage “fruit formation: beginning of bunch formation” (BBCH 77) on weakened leaves. In red varieties of the Western European eco-geographical group (Merlo, Cabernet Sauvignon) and in a variety from the Black Sea basin, convar *pontica* Negr. (Saperavi), the severity of the disease was the least and comprised 0–0.7% and 0.9%, respectively. Moreover, on these varieties, spotting was most often developing on aging, weakened, or damaged leaves (for example, as a result of sunburn), while the severity of the disease was beginning much later than on interspecies hybrids.

### 2.2. Morphological Characterization of Isolates

During the period of 2020–2021, we isolated from grapevine leaves micromycetes belonging to nine genera: *Alternaria*, *Fusarium*, *Phomopsis*, *Aspergillus*, *Cladosporium*, *Mucor*, *Penicillium*, and *Trichoderma*. Based on morphological characteristics, the largest number of isolates (433 isolates in total) were assigned to the genus *Alternaria*.

**Figure 2:**
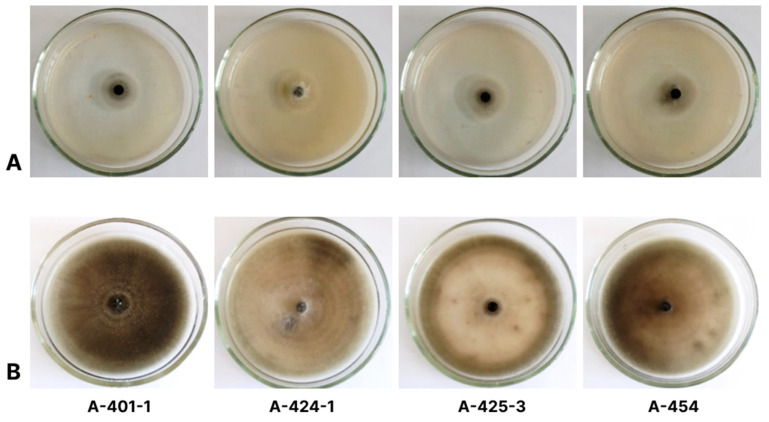
*Alternaria* sp. isolates on PCA medium: (**A**) 3 dpi and (**B**) 10 dpi.

On the PCA nutrient medium, colonies of *Alternaria* sp. isolates had a regular shape with a smooth villous edge. After 7 days post-inoculation (dpi), the average colony diameter was 67 to 81 mm. The mycelium was loose, with a flat profile, from 0.2 to 1.5 mm in height. Its color varied from light gray to dark gray with a greenish tint (Figure 2). The center of the colony was darker.

**Figure 3:**
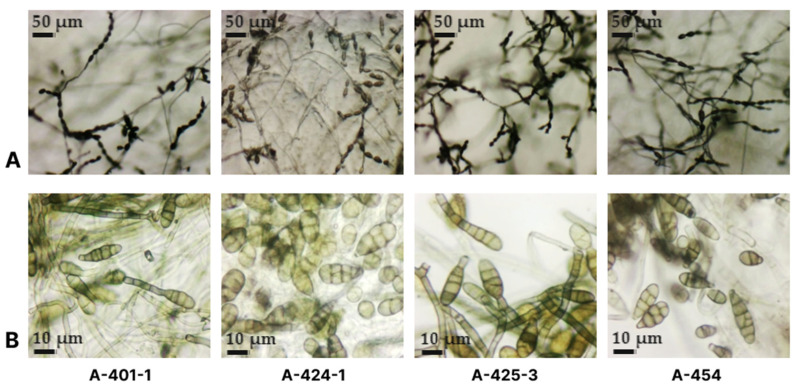
Sporulation pattern of *Alternaria* sp. isolates of 10 dpi (**A**) and conidia (**B**) on PCA medium.

The typical sporulation pattern comprised chains of conidia of varying length (Figure 3A). Individual chains from the initial to the youngest spore had 9–18 conidia. The first conidium on a conidiophore was narrowly elliptical, straight, often unevenly sided, and rarely strongly elongated in shape. The conidia that formed later in the chain were generally wider and shorter, ovoid, elliptical, and pear-shaped with well-developed secondary conidiophores. Conidia had 0–4 longitudinal septa and 1–5 transverse ones (Figure 3B). Their color was brown or dark brown. The size of the mature conidia was 14.9–32.7 × 5.5–12.2 µm. The conidiophores were gray-brown, straight or curved, and branched, with noticeable spore markings. Based on the morphological characteristics of the microstructures, all isolates were identified as *A. alternata* [21].

For most of the tested nutrient media, the growth parameters of *Alternaria* sp. isolates were similar and were characterized by a radial growth rate of 0.26–0.54 mm/hour, the rapid development of vegetative mycelium, and active sporulation, which made it difficult to analyze the sporulation pattern and location of primary conidiophores (Supplementary Figure S2). On the PCA nutrient medium, a fairly high growth rate and moderate formation of conidia were observed, which facilitated microscopic examination of the isolates. The highest radial growth rate was on the TPA medium, and the lowest was on WA and PAVE. On the PA, PACA, and PCA nutrient media, the colonies had a dark gray color, on CMA, OAT and HA they were gray, on V-8 they were black, and on TPA medium they were light gray, almost white (Supplementary Figure S3). Thus, PCA was optimal for analyzing isolates of the *Alternaria* genus.

### 2.3. Pathogenicity Test

After the inoculation of grapevine leaf discs with *Alternaria* sp. isolates, the pathogen was re-isolated from the resulting necroses, thereby completing Koch’s postulates. The isolates were identified as *Alternaria* sp. and corresponded to the original ones.

**Figure 4:**
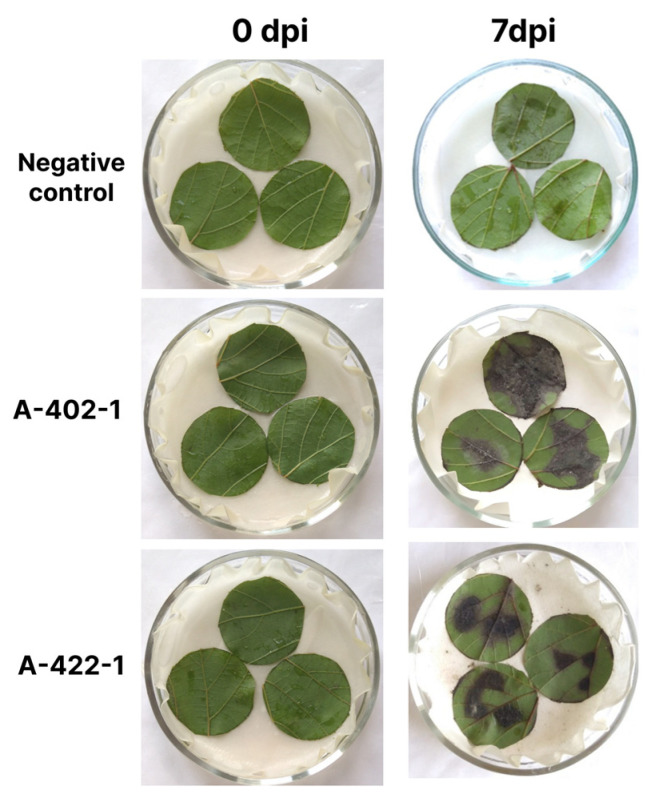
In vitro inoculation of grapevine leaves of the Bianca variety with *Alternaria* sp. isolates.

The virulence of isolates of *Alternaria* spp. varied after inoculation into five grapevine varieties (Table 2, Figure 4). The most virulent isolate A-429-2 formed a necrotic zone occupying more than 50% of the leaf disc area on all tested varieties. Isolate A-402-1 was highly virulent on the Euro-American hybrids Avgustin, Levokumskij, and Bianka and weak on the hybrids Moldova and Pervenets magaracha. Isolates A-422-1 and A-447 were weakly or moderately virulent only to the hybrids Avgustin, Levokumskij, and Bianka, while isolates A-443 and A-448 were weakly or moderately virulent to four of the five tested varieties. Two of the tested isolates (A-422-2 and A-426-2) were nonpathogenic on all varieties.

Thus, the isolates of *Alternaria* sp. were characterized by the varying degree of pathogenicity in relation to grapevine varieties and represented a mosaic population.

### 2.4. Molecular Genetic Analysis

Due to the high plasticity of the morphological characteristics of *Alternaria* fungi, the analysis of only the morphological and cultural features is often insufficient for their identification. A total of 35 representative isolates from different grapevine varieties with signs of necrotic leaf spot were selected for multilocus typing (Supplementary Table S1). All *Alternaria* sp. isolates yielded PCR products of the expected size for the ITS, *Alt a1*, *tef1*, and *gapdh* loci. For six isolates, the amplification of the *tub* gene with the T1/T22 primer pair was unsuccessful; therefore, an additional T1/Beta-tub-2 pair was used (Supplementary Table S2). As a result, we amplified and sequenced five markers for all selected strains. The aligned sequences of *Alt a1* (445 bp), *gapdh* (516 bp), ITS (513 bp), *tef1* (569 bp), and *tub* (954 bp) contained twenty-eight, one, zero, five, and twelve variable sites, respectively (Table 3). Among the five genes analyzed in our sample, the *Alt a1* gene was the most variable: the percentage of polymorphic sites comprised 6.3%.

The analysis of sequences of *Alt a1*, *gapdh*, and ITS markers in an expanded sample which additionally included 90 representative isolates from the *Alternaria* section showed a similar trend; the *Alt a1* gene was the most polymorphic (20.7% of polymorphic sites) (Table 4).

**Figure 5:**
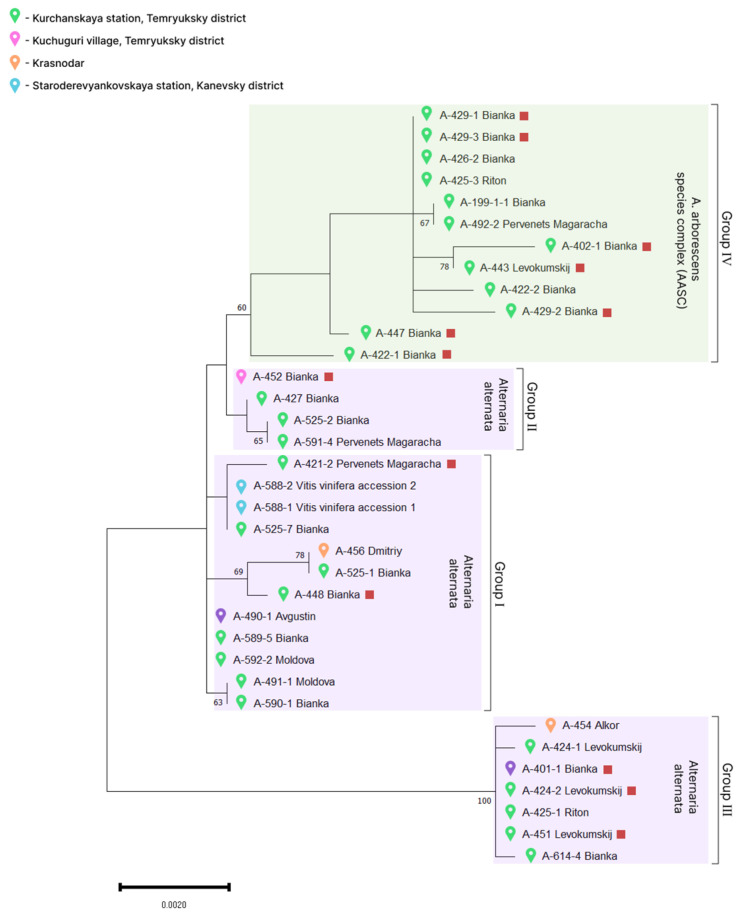
Phylogenetic tree based on the sequences of *Alt a1*, *gapdh*, ITS, *tef1*, and *tub* markers of 35 *Alternaria* sp. isolates from grapevine samples with leaf spot symptoms. Group names are given taking into account clustering in the dendrogram with representative isolates. The strains that showed pathogenicity in the grape test are indicated by a red square. The tree was constructed in MEGA 11 using the Maximum Likelihood method and the Tamura-Nei model with 1000 bootstrap replicates. Only bootstrap values greater than 60 are shown.

In the dendrogram constructed using concatenated nucleotide sequences of *Alt a*1, *tef1*, *gapdh*, ITS, and *tub* (2949 bp) of *Alternaria* sp. isolates from grapevines, two clades were observable, in one of which three more subclades can be distinguished (Figure 5). Isolates collected in the same locations from the same grapevine varieties clustered throughout the tree.

**Figure 6:**
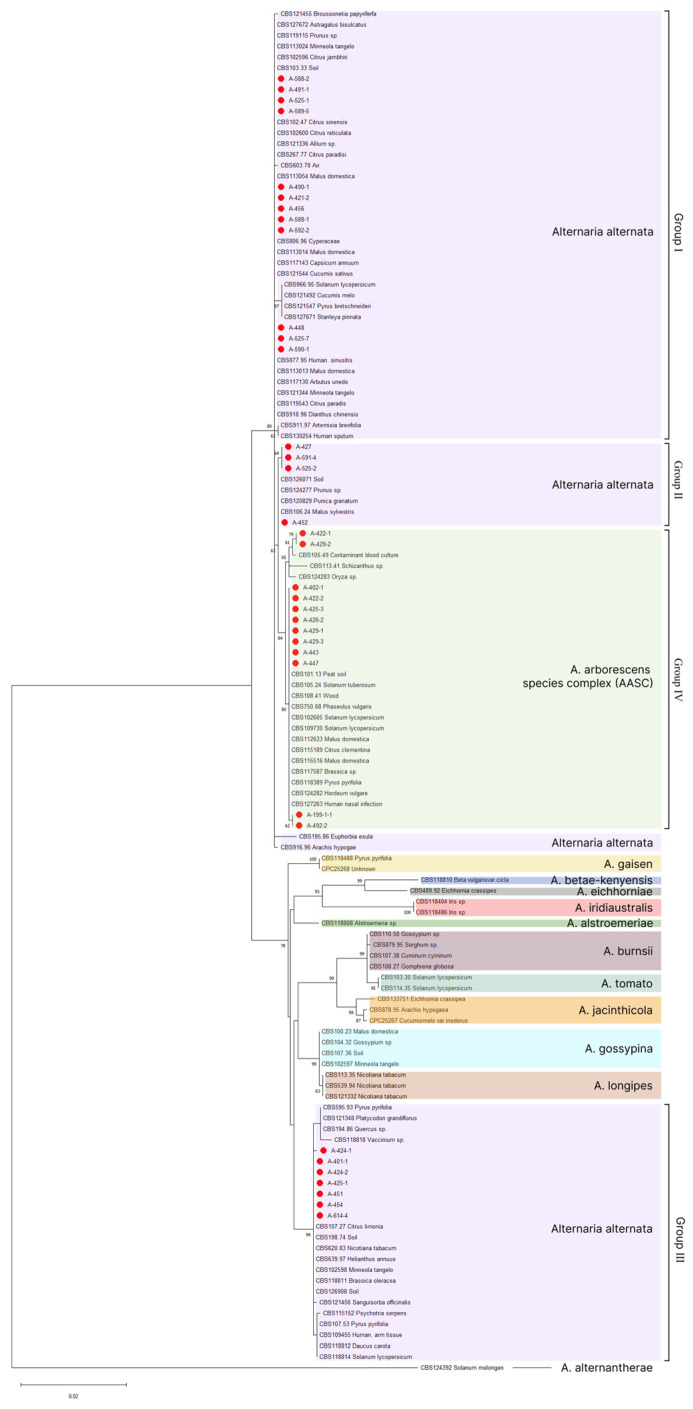
Phylogenetic tree constructed by the Maximum Likelihood method (1000 bootstrap replicates) using concatenated *Alt-a*1, *gapdh*, and ITS marker sequences of *Alternaria* sp. isolates from grapevines obtained in this study (red dot) and representative isolates of the *Alternaria* section. The tree was rooted with *A. alternantherae* (CBS 124392). Only bootstrap values greater than 60 are shown.

Due to the lack of reference sequences of the required length for the *tub* and *tef1* genes, the tree with representative isolates of the *Alternaria* section was constructed using concatenated sequences (1476 bp long) of three markers: *Alt a1*, *gapdh*, and ITS. A phylogenetic analysis allowed us to distinguish 15 clades in the dendrogram (Figure 6). Eight of them were formed by individual species (*A. alstroemeriae*, *A. betae-kenyensis*, *A. eichhorniae*, *A. gaisen*, *A. iridiaustralis*, *A. jacinthicola*, *A. longipes*, and *A. tomato*). Three more clades were represented by morphospecies united into an *A. burnsii*, *A. gossypina*, and *A. arborescens* species complex, according to the classification proposed by Wounderberg et al. [28]. The morphospecies synonymized under the name *A. alternata* were divided into two branches, forming three additional clades within one of them. The isolates from grapevines were distributed into four groups corresponding to four subclades in the dendrogram constructed using five markers. Three groups clustered together with *A. alternata* isolates in different branches of the tree (Figure 6, Groups I–III). Group IV clustered together with isolates of the *A. arborescens* species complex. The dendrogram branch uniting isolates of the *A. arborescens* species complex was located within the *A. alternata* clade, but even with a data set limited to three genes, it was still forming a separate cluster.

In the dendrogram constructed using the nucleotide sequences of the *Alt a1* gene, the same 13 clades corresponding to different species in the *Alternaria* section (Supplementary Figure S4) can be distinguished; *A. longipes* and *A. gossypina* clustered together. Isolates from grapevines clustered together with the same representative isolates of the *A. alternata* and *A. arborescens* species complex.

The taxonomic allocation of isolates from grapevines and the identification of species within the *Alternaria* section based on the phylogenetic trees constructed using the *gapdh* and ITS nucleotide sequences were, as expected, difficult (Supplementary Figures S5 and S6). Based on the *gapdh* sequence, it was possible to distinguish *A. burnsii*, *A. tomato*, *A. jacinthicola*, *A. iridiaustralis*, *A. eichhorniae*, and *A. betae-kenyensis*. In the dendrogram constructed using the ITS marker, the species *A. iridiaustralis*, *A. eichhorniae*, and *A. betae-kenyensis* were clearly distinguishable. The conservativeness of regions of these two genes can be used to identify *Alternaria* micromycetes to the genus.

Thus, the analysis of the *Alt a1*, *gapdh*, and ITS sequences confirmed that all micromycete isolates from grapevine plants with symptoms of leaf spot belonged to the *Alternaria* section. A total of 23 isolates were found to cluster together with the *A. alternata* morphospecies and another 12 strains clustered with morphospecies of the *A. arborescens* species complex.

### 2.5. Screening of Fungicides and Antagonists Against Alternaria sp.

**Figure 7:**
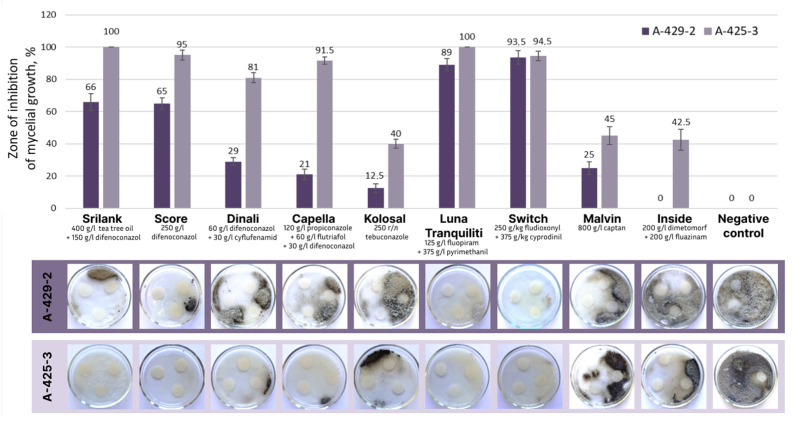
Fungicidal activity of chemical preparations from different groups against the *Alternaria* sp. isolates A-425-3 and A-429-2.

As a result of studying the fungicidal activity of preparations with active substances from the group of triazoles, preparations containing difenoconazole (trade names Score, Srilank, Dinali, and Capella) were found to possess a high fungicidal activity against isolate 425-3 (efficiency of 81–100%) and isolate A-429-2 (efficiency of 21–66%) (Figure 7, Supplementary Figure S7). Among them, Srilank and Score were the most efficient. The efficiency of a preparation containing tebuconazole (trade name Kolosal) was at the level of 12.5–40%, while Domark with the same active substance did not show fungicidal activity against the studied isolates.

The active substances from the group of strobilurins, azoxystrobin (trade names Quadris and Intrada) and kresoxim-methyl (trade name Stroby), did not have fungicidal activity against isolates A-425-3 and A-429-2. A combination of the active substances pyraclostrobin and metiram was found to possess a low fungicidal activity (mycelial growth inhibition zone of 2–4 mm) (trade name Cabrio Top) (Supplementary Figure S8).

An active substance from the group of dithiocarbamates (mancozeb), both when used alone and in combination with an active substance from the group of phenylamides (mefenoxam), had a weak fungicidal activity against the studied isolates (Supplementary Figure S9).

The fungicidal activity of the tested copper-based compounds was not detected (Supplementary Figure S10).

Among all analyzed chemical groups, the highest fungicidal activity was demonstrated by preparations containing anilinopyrimidine in combination with benzamide and phenylpyrrole (Supplementary Figure S11). A combination of the active substances pyrimethanil and fluopyram (trade name Luna Tranquility) showed an efficiency at a level of 89–100%, while a combination of cyprodinil and fludioxonil (trade name Switch) showed efficiency at a level of 93.5–94.5% (Figure 7).

The efficiency of the active substance captan from the group of phthalimides (trade name Malvin) was at the level of 25–45% against both isolates. A combination of the active substances dimethomorph and fluazinam (trade name Inside) was efficient against only one isolate A-425-3 at the level of 40%. The active substances from other groups did not show fungicidal activity against the studied isolates (Supplementary Figure S11).

Previously, we performed an assessment of the antagonistic activity of 15 *Trichoderma* sp. strains against the *Alternaria* sp. isolate 425-3 using the double-culture method [51]. To demonstrate the possibility of controlling *Alternaria* sp. not only using chemical pesticides, in this work we compared the previously obtained data on the antagonistic activity and determined the PIRG for promising strains of *Trichoderma* sp. PIRG was found to range from 35 to 92% (Table 5).

The *Trichoderma* strains T-213, T-338, T-404/1, T-503, and F-838 showed antimycotic activity above 80%. Strains T-404/1 and F-838, which demonstrated antimycotic activities of 91.3 and 92.1%, respectively, can be used as antagonists in the development of methods for the integrated control of the spread of *Alternaria* sp. on grapevines.

## 3. Discussion

In this study, we provide, for the first time, data on a large-scale leaf spot symptom observation of grapevines affected by fungi of the genus *Alternaria*. The first symptoms of leaf spot have been identified by us earlier [50]. To date, this disease has also been detected in Italy [52]. The increase in the spread of *Alternaria* leaf spot of grapevines may indicate the active biological progress of fungi of the genus *Alternaria*. Large surfaces of host plants with low genetic diversity are known to support large populations of pathogens with high evolutionary potential, which allows them to adapt quickly to new hosts and changing environmental conditions [4,53]. Therefore, a possible reason for the emergence of pathogenic strains of *Alternaria* spp. in vineyards is the increased influence of anthropogenic and abiotic factors. Monoculture and climate changes also contribute to the active expansion of the ranges of new pathogenic species.

To define the optimal medium for analyzing the *Alternaria* spp. isolates, ten media were tested. Some of them were previously used for the cultivation of *Alternaria* spp. According to the growth rate and the sporulation, the PCA medium was optimal. This medium is one of the recommended media for the analysis of *Alternaria* species [21].

The high similarity of the morphological and cultural features of *Alternaria* spp. isolates from grapevine necessitates the use of additional methods for the taxonomic identification of isolates. The results of the multilocus typing of *Alternaria* spp. isolates indicate the presence of at least four phylogenetically distinct groups belonging to the clades of the *A. alternata* and *A. arborescens* species complex. It should be noted that the combined sequences of the three genes (*Alt a1*, *gapdh*, and ITS) were sufficient to distinguish the species of the *Alternaria* section, with the *Alt a1* gene being the most informative in determining the phylogenetic relationships of the isolates, which is consistent with the results of the *Alt a1* gene study by Hong et al. [38]. It was found that some of the reference strains of *A. alternata*, as well as seven *Alternaria* sp. isolates from grapevines, formed a clade located at a distance from the other strains of *A. alternata*. The allocation of these strains to a separate *A. alternata* group is consistent with the study by Woudenberg et al. [28], where these isolates were also grouped separately, but as a part of the general clade of *A. alternata*. There are certain difficulties in selecting a sufficient number of representative isolates in GenBank when using the *tef1* and *tub* genes for multilocus typing. Therefore, the continuation of genetic studies of *Alternaria* spp. and the development of a unified set of loci and primers for typing would significantly facilitate the identification of micromycetes.

Since the *Alternaria* spp. isolates that caused in vitro necrosis on grapevine leaf discs were located in each of the four groups on the dendrogram, no relationship was found between phylogenetic position and pathogenicity. In addition, there was no relationship between the clustering of isolates on the dendrogram, the host plant variety, and the sample location. This indicates a high intraspecies heterogeneity of *Alternaria* spp. isolates.

In this study, we identified efficient chemical fungicides and promising biological agents for use in integrated schemes for the control of *Alternaria* leaf spot of grapevine. Of all of the analyzed groups of fungicides, the highest fungicidal activity was demonstrated by preparations containing substances from the group of anilinopyrimidines in combination with benzamide or phenylpyrrole (pyrimethanil with fluopyram and cyprodinil with fludioxonil) and from the group of triazoles (difenoconazole and difenoconazole with tea tree oil). The efficiency of fungicides including these active substances has also been previously demonstrated against *A. solani* which causes brown leaf spotting of potatoes and early blight of tomatoes, as well as against *A. alternata* which causes postharvest fruit rot of blueberries [54,55,56]. In our study, isolates of *Alternaria* spp. showed different levels of susceptibility to the same preparations. Therefore, it is necessary to conduct a preliminary screening of fungicides from different chemical groups when choosing means to control *Alternaria* leaf spot of grapevines. Monitoring the efficacy of preparations is also important in terms of controlling the development of resistance to fungicides and detecting early shifts in the sensitivity to them in *Alternaria* spp.

The introduction of alternative control means, such as biological agents, not only improves the environmental safety of crops, but also slows down the process of reduction in sensitivity to chemical fungicides. In this study, we provide data on 15 *Trichoderma* sp. strains that exhibit antagonistic activity against *Alternaria* sp. The PIRG values that we established for *Trichoderma* sp. strains T-404/1 and F-838 (~90%) exceed the previously published PIRG values for *Trichoderma harzianum*, *Trichoderma longibrachiatum*, and *Trichoderma viride* (~70–75%) used against *A. porri* and *A. alternata* [57,58]. *Trichoderma* sp. strains T-404/1 and F-838 can be used as biological agents in the development of biofungicides that can be included in an integrated protection system of grapevine against leaf spot caused by *Alternaria* spp.

These results expand our knowledge of the biodiversity of *Alternaria* spp. fungi and can be used in further studies and then to limit the spread of *Alternaria* leaf spot of grapevines. Further studies shall be aimed at clarifying the phylogenetic relationships of the identified *Alternaria* spp. isolates using a larger number of molecular markers or whole genome sequencing.

## 4. Materials and Methods

### 4.1. Field Survey and Morphological Characterization of Alternaria spp.

Leaf spot symptoms observation was carried out in June and July 2020 and 2021 in commercial vineyards with a total area of 670 hectares located in the Krasnodar Krai (Russia) (Supplementary Figure S12). The incidence and severity of leaf spot was assessed during the period of intensive infection development in the growth stages of berry growth and bunch formation (BBCH 71–79) by route surveys.

The incidence of the disease was calculated as the number of diseased plants divided by the total number of assessed plants:

I = n/N · 100%,(1)

in which I is the incidence of disease (%), n is the number of diseased plants, and N is the total number of assessed plants (diseased and healthy).

The severity of the disease was assessed based on a five-point scale (0—no disease symptoms; 1—the appearance of a minimal amount of symptoms on the plant, 2—up to 10% of the leaf surface is affected; 3—up to 11–25% of the leaf surface is affected; 4—up to 26–50% of the leaf surface is affected; and 5—more than 50% of leaf surface is affected). The severity of the disease was calculated according to the following equation:

S = ∑(a · b) · 100%/N · K,(2)

in which S is the disease severity (%), a is the number of diseased plants and b is the corresponding damage score in points, N is the total number of assessed plants, and K is the highest score on the scale.

Grapevine leaves with necrotic spots were collected and transported in individual bags to the laboratory. An affected leaf fragment with an area of 3–5 cm^2^ was disinfected with 70% ethanol for 40 s, treated with 1% sodium hypochlorite for 60 s, washed three times with sterile distilled water and dried with filter paper. Then, it was placed on the PDA nutrient medium supplemented with 100 mg/L streptomycin sulfate and incubated for 4–7 days at 26 °C with a 12 h photoperiod. Hyphal fragments were isolated, grown, and stored at +4 °C [59].

For identification, the isolates were cultured on potato carrot agar (PCA) for 10 days, after which their morphological and cultural properties were determined as described previously [21]. The radial growth rate and growth coefficient were determined on the 7th day using formulas 3 [60] and 4 [61] as follows:

Kr = (R_1_ − R_0_)/(t_1_ − t_0_), (3)

where Kr = the radial growth rate of a colony, mm/hour; R_1_ = colony radius at time t_1_; and R_0_ = colony radius at t_0_.

GC = D × h × g/t,(4)

in which GC—growth coefficient; D—colony diameter, mm; h—mycelium height, mm; g—colony density, point; and t—colony growth time, days.

The parameters of the growth of *Alternaria* isolates were studied on nutrient media: potato carrot agar (PCA) [62], vegetable juice agar (V-8) [62], corn meal agar (CMA) [63], potato agar (PA) [63], oatmeal agar (OAT) [63], water agar (WA) [64], tomato pulp agar (TPA) [65], potato agar with citric acid (PACA), potato agar with valerian root extract (PAVE), and hay infusion agar (HA) (Supplementary Table S3). Parameters for incubation were as described above.

### 4.2. Pathogenicity Test

To confirm Koch’s postulates, leaf discs of five grapevine varieties, including the variety from which the isolate was obtained, were inoculated. Fifteen isolates of *Alternaria* spp. were grown on PCA for 14 days, then a suspension of 1 × 10^6^ conidia/mL was prepared. Grapevine leaf discs with a diameter of 40 mm were disinfected in a 5% NaOCl solution for 5 min, washed with sterile distilled water and placed on wet filter paper in Petri dishes. The disk surface was inoculated with a spore suspension and incubated for 7 days, after which the size of the necroses was measured. Isolates causing necroses with a size of up to 10% of the leaf disk surface were considered weakly virulent, those with a size of 10–50% of the disk area were considered moderately virulent, and those with a size of more than 50% were considered highly virulent.

### 4.3. DNA Extraction, Sequencing, and Phylogenetic Analysis

Representative *Alternaria* spp. isolates from different locations and cultivars and with different pathogenicity were selected for molecular analysis. DNA extraction from the mycelium of pure fungal cultures was performed in accordance with the method of Al-Sanae et al. [66]. The quality of the extracted DNA was verified using a BioSpectrometer kinetic spectrophotometer (Eppendorf, Hamburg, Germany) and electrophoresis on a 1.2% agarose gel. The extracted DNA was stored at −20 °C. The amplification of the internal transcribed spacer (ITS), glyceraldehyde-3-phosphate dehydrogenase (*gapdh*), *Alternaria* allergen a1 (*Alt a1*), β-tubulin (*tub*), and translation elongation factor (*tef1*) markers was performed using the following specific primers (Supplementary Table S2): ITS1/ITS4 [67], gpdF/gpdR [68], Alt-For/Alt-Rev [38], T1/T22 [69], T1/Beta-tub-2 [70], and Alt-tef1/Alt-tef2 [71]. The amplification products were visualized on a 1.2% agarose gel and column purified using the Cleanup Standard kit (Evrogen, Moscow, Russia).

PCR amplification products were sequenced by bidirectional Sanger sequencing on an ABI PRIZM 3730 automated sequencer using the Cycle Sequencing Kit BigDye Terminator v3.1 (Thermo Fisher Scientific, Waltham, MA, USA) according to the manufacturer’s instructions. The resulting nucleotide sequences were analyzed using the NCBI BLASTn, Finch TV 1.4.0 (Geospiza Inc., Seattle, WA, USA) and MEGA11 software [72]. The sequences were submitted to GenBank (Supplementary Table S4). Multiple alignments of DNA sequences were performed for each marker. Total, variable, parsimony-informative, and singleton sites were analyzed using MEGA11 software. The number of polymorphic sites was calculated as the percentage of variable sites in the total number of sites.

For phylogenetic analysis, the nucleotide sequences of five genes were trimmed and merged into a single sequence. The analysis included representative sequences from previous studies [26,27,28]. The best model of DNA sequence evolution was found using the MEGA11 software. The multigene phylogenetic tree based on the nucleotide sequences of *Alt a1*, *gapdh*, and ITS, as well as trees based on the sequences of the *Alt a1* and *gapdh* loci, was constructed using the Kimura 2-parameter model. The Tamura-Nei model was used to create the five-marker phylogenetic tree. The phylogenetic tree based on the ITS sequences was constructed using the Jukes-Cantor model. All dendrograms were constructed using the Maximum Likelihood method with 1000 bootstrap replicates. As an outgroup in all generated trees, we used homologous sequences of the *Alt a1*, *gapdh*, and ITS of *Alternaria alternantherae* (CBS 124392), the type species of the section *Alternantherae*. The bootstrap support included 1000 replicates.

### 4.4. Screening of Alternaria sp. Sensitivity to Fungicides and Assessment of Antagonistic Activity of Trichoderma sp.

The activity of chemical fungicides against pathogenic isolates of *Alternaria* sp. A-429-2 and A-425-3 was analyzed by the disk diffusion method [73]. The list of fungicides tested is presented in Supplementary Table S5. To prepare working solutions, weighted amounts of fungicides were dissolved in sterile distilled water to a concentration corresponding to the application rate of the preparation [74]. The PCA medium was inoculated with the spore suspension prepared as described previously, and disks moistened with a freshly prepared fungicide solution were placed on it. Disks moistened with sterile distilled water were used as a control. On the 10th day, the diameter of the fungal growth inhibition zone was measured. The drug efficiency was assessed by the diameter of the fungal growth inhibition zone at 10 dpi. The experiments were carried out in 4 replicates.

To assess the antagonistic activity of 15 *Trichoderma* sp. isolates against *Alternaria* sp. isolates, we calculated the percentage of the inhibition of radial growth (PIRG) using the following formula [75]:

PIRG= (R1 − R2)/R1 × 100, (5)

in which R1 is the radial growth of the *Alternaria* sp. isolate without the *Trichoderma* sp. isolate, and R2 is the radial growth of the *Alternaria* sp. isolate with the *Trichoderma* sp. isolate.

## Figures and Tables

**Table 1:** Incidence and severity of leaf spot on different grapevine varieties.

	Grapevine Varieties	Genotype	Incidence of Disease, %	Severity of the Disease, %
Euro-American hybrids	Bianka	(*V. vinifera* + *V. labrusca* + *V. riparia* + *V. rupestris* + *V. berlandieri* + *V. aestivalis* + *V. cinerea*) × *V. vinifera*	81.2 ± 17.1	58.9 ± 7.8
	Moldova	(*V. vinifera* × (*V. vinifera* + *V. labrusca* + *V. riparia* + *V. rupestris* + *V. berlandieri* + *V. aestivalis* + *V. cinerea*)	30.4 ±11.4	22.4 ± 2.7
	Avgustin	*V. vinifera* × (*V. vinifera* + *V. labrusca* + *V. rupestris* + *V. berlandieri* + *V. lincecumii*)	38.0 ± 16.9	24.4 ± 4.1
	Dunavski Lazur	*V. vinifera* × (*V.vinifera* + *V. labrusca* + *V. rupestris* + *V. berlandieri* + *V. lincecumii*.)	43.8 ± 5.4	22.4 ± 3.0
	Pervenets Magaracha	*V. vinifera* × (*V. vinifera* × (*V. vinifera* + *V. riparia* + *V. rupestris*))	55.2 ± 12.7	28.8 ± 5.1
	Citronny Magaracha	*V. vinifera* +*V. vinifera* + *V. labrusca*	15.1 ± 6.3	4.8 ± 1.8
	Levokumskij	*V. vinifera* + *V. labrusca*	89.9 ± 9.8	61.9 ± 4.8
	Dekabrskii	*V. vinifera* × (*V. vinifera* + *V. labrusca* + *V. rupestris* + *V. berlandieri* + *V. lincecumii*)	29.2 ± 3.7	14.7 ± 2.9
	Doina	*V. lincecumii* +*V. rupestris* + *V. vinifera*	34.2 ± 8.3	17.1 ± 4.4
Euro-Amur hybrids	Kunleany	(*V. vinifera* × *V. amurensis*) × *V. vinifera* convar. *boreali-africana*	0	0
	Bruskam	*V. vinifera* × *V. labrusca* × *V. amurensis*	0	0
	Amur	*V. vinifera* convar. *orientali-mediterranea* × *V.amurensis* × *V. vinifera*	0	0
	Cristal	*V. vinifera* × *V. amurensis*	5.1 ± 1.5	3.5 ± 0.9
	Vostorg	*V. vinifera* × *V. amurensis*	7.4 ± 4.1	3.1 ± 1.7
European varieties	Risling Rejnski	*V. vinifera* convar *occidentalis* subconvar. *gallica*	14.3 ± 8.1	5.2 ± 1.8
	Sauvignon Blanc		26.4 ± 6.1	9.0 ± 1.0
	Aligote		11.2 ± 1.8	2.5 ± 0.7
	Chardonnay		10.5 ± 1.2	0.8 ± 0.2
	Pinot blanc		5.3 ± 0.9	2.0 ± 0.3
	Muller Thurgau		29.1 ± 5.7	9.2 ± 1.2
	Traminer Pink		0	0
	Cabernet Sauvignon		0	0
	Merlo		3.7 ± 0.6	0.7 ± 0.1
	Saperavi	*V. vinifera* convar *pontica* subconvar. *georgica*	2.3 ± 0.2	0.9 ± 0.1

**Table 2:** Pathogenicity and virulence of *Alternaria* sp. isolates using different grapevine varieties.

Isolate	Grapevine Varieties				
	Avgustin	Levokumskij	Bianka	Moldova	Pervenets Magaracha
A-401-1	+	+	+	+	+
A-402-1	+++	+++	+++	+	+
A421-2	+	+	+	+	+
A-422-1	++	++	++	-	-
A-422-2	-	-	-	-	-
A-424-2	++	++	++	++	-
A-426-2	-	-	-	-	-
A-429-1	+	+	+	+	-
A-429-2	+++	+++	+++	+++	+++
A-429-3	+	+	+	+	+
A-443	+	+	++	++	-
A-447	+	+	+	-	-
A-448	++	+	++	-	++
A-451	++	+	+	+	++
A-452	+	+	+	+	+

Note: “-”–no necrosis; “+”–small point necrosis (up to 10% of the surface area of leaf discs is affected); “++”–necrosis of 10–50% of the area of leaf discs; “+++”–necrosis of more than 50% of the area of leaf discs.

**Table 3:** Analysis of nucleotide sequence diversity of *Alt a1*, *gapdh*, ITS, *tef1*, and *tub* markers in a sample of 35 *Alternaria* sp. isolates from grapevines.

Gene Sequence	Total Sites	Constant Sites	Variable Sites	Parsimony-Informative Sites	Singleton Sites	Polymorphic Sites (%)
*Alt a1*	445	416	28	28	0	6.3
*gapdh*	516	515	1	1	0	0.2
ITS	513	506	0	0	0	0
*tef1*	569	564	5	3	2	0.9
*tub*	954	942	12	9	3	1.3

**Table 4:** Analysis of nucleotide sequence diversity of *Alt a1*, *gapdh*, ITS, and tub markers in a sample of 125 *Alternaria* sp. isolates.

Gene Sequence	Total Sites	Constant Sites	Variable Sites	Parsimony-Informative Sites	Singleton Sites	Polymorphic Sites (%)
*Alt a1*	445	353	92	69	23	20.7
*gapdh*	506	464	42	29	13	8.3
ITS	479	470	5	3	2	1.0

**Table 5:** Percentage of inhibition of radial growth of *Alternaria* sp. isolates by *Trichoderma* sp.

Index	Strains of *Trichoderma* sp.														
	Tk-1	T1	T1	T3	T4	T5	T-213	T338	T-404/1	T-441/1	T-503	F-218	F-219	F-294	F-838
PIRG *, %	54.6	52.7	61.8	58.3	70.6	59.7	86.0	87.4	91.3	79.5	82.8	34.9	59.8	71.7	92.1

* Least Significant Difference (05) = 0.78405.

## Data Availability

Sequences were deposited in GenBank under the accession numbers PQ583906-PQ584045, PQ590405-PQ590439.

## Supplementary Materials

The following supporting information can be downloaded at: https://www.mdpi.com/article/10.3390/plants13233335/s1, Figure S1: 1. Symptoms of leaf spot disease on Euro-American hybrids and European varieties of grapevines; Figure S2: Growth parameters of *Alternaria* sp. isolates on different nutrient media; Figure S3: Colonies of Alternaria sp. isolate A-203-7 on different nutrient media (7 dpi); Figure S4: Phylogenetic tree constructed using *Alt-a1* marker sequences of *Alternaria* spp.; Figure S5: Phylogenetic tree constructed using *gapdh* marker sequences of *Alternaria* spp. isolates; Figure S6: Phylogenetic tree constructed using ITS marker sequences of *Alternaria* spp. isolates; Figure S7: Fungicidal activity of chemical preparations substances from the group of triazoles; Figure S8: Fungicidal activity of chemical preparations substances from the group of strobilurins; Figure S9: Fungicidal activity of chemical preparations substances from the group of dithiocarbamates; Figure S10: Fungicidal activity of copper-based chemicals; Figure S11: Fungicidal activity of chemicals from other chemical groups; Figure S12: Map of the Krasnodar Territory of Russia; Table S1: Basic information about representative strains of *Alternaria* spp. for multilocus typing; Table S2: List of the PCR primers and programs; Table S3: Nutrient media for the cultivation of *Alternaria* sp.; Table S4: Sequences of Alta 1, ITS, gapdh, tef1, tub uploaded to GenBank with their identifiers; Table S5: List of fungicides used for testing on pathogenic isolates of *Alternaria* sp.
