# Supplementary material for: Leaf Spot Caused by Alternaria spp. Is a New Disease of Grapevine
Source: Plants (Basel). 2024 Nov 28;13(23):3335. doi: 10.3390/plants13233335 (PMC11644299; doi:10.3390/plants13233335)
Supplement: Supplementary file 1 [file plants-13-03335-s001.zip › Supplementary_Figures new.pdf]

Leaf spot caused by *Alternaria* sp. is a new disease of grapevine

Evgeniya Yurchenko [1], Daria Karpova [1, 2], Margarita Burovinskaya [1] and Svetlana Vinogradova [2, *]

[1] North Caucasian Federal Scientific Center of Horticulture, Viticulture, Wine -Making, 40 Years of Victory Street, Build. 39, 350901 Krasnodar, Russia

[2] Skryabin Institute of Bioengineering, Research Center of Biotechnology of the Russian Academy of Sciences, Leninsky Prospect, 33, Build. 2, 119071 Moscow, Russia

* Correspondence: coatprotein@bk.ru

Supplementary Materials

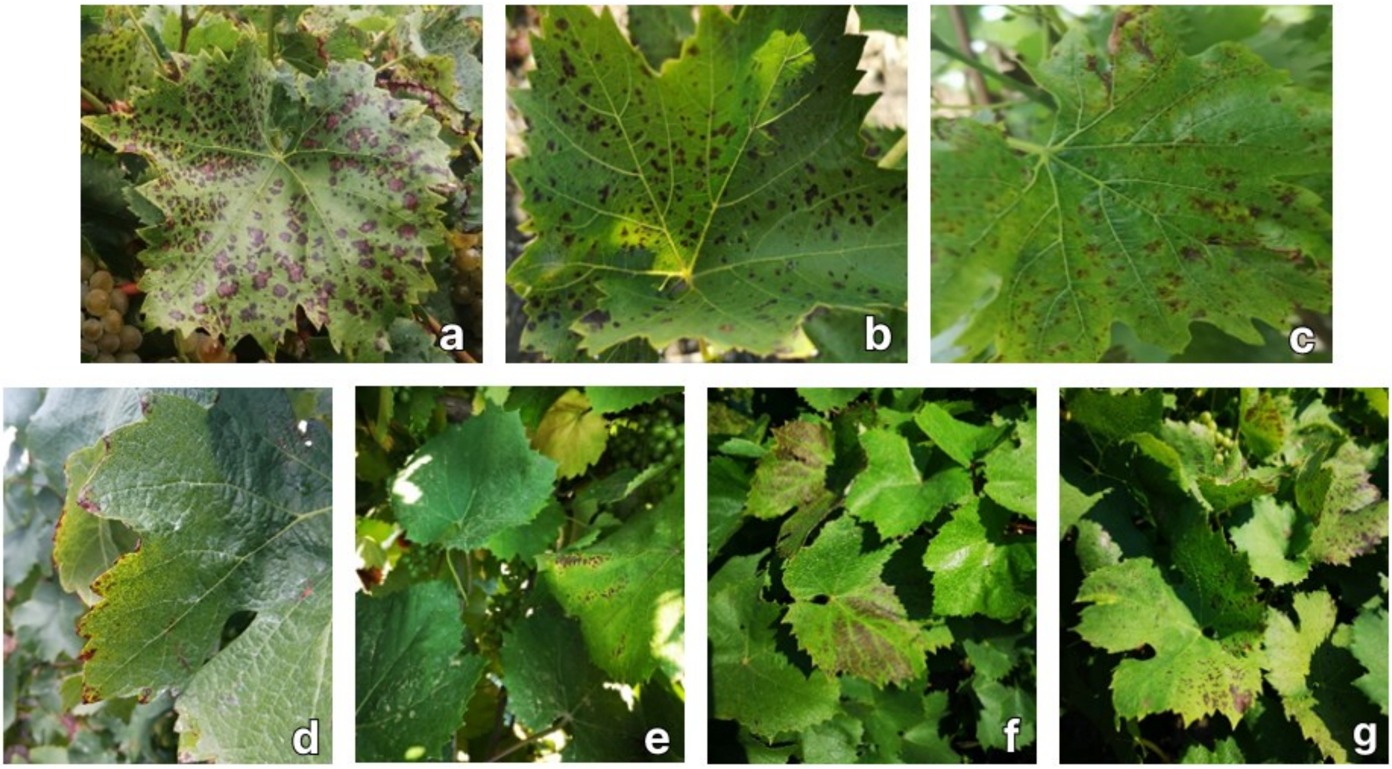

Supplementary Figure S1. Symptoms of Leaf spot disease on euro-american hybrids (a – Bianka; b - Pervenets magaracha; c – Avgustin) and european varieties of grapevine (d – Sauvignon blanc, e – Riesling reinskii, f – Chardonnay, g – Pinot blanc).

Nutrient medium	Isolates	Colony diameter, cm, 7dpi	Kr , mm/hour	GC
PA	A-198-1-1	5,4±0,5	0,32	0,23
	203-7	4,4±0,8	0,26	0,19
PACA	198-1-1	5,5±0,4	0,33	0,24
	203-7	6,2±0,15	0,37	0,26
PCA	198-1-1	5,4±0,2	0,32	0,23
	203-7	4,9±0,4	0,29	0,21
OAT	198-1-1	6,0±0,05	0,36	0,86
	203-7	4,9±0,5	0,29	0,55
PAVE	198-1-1	3,0±0,3	0,18	0,04
	203-7	4,4±0,4	0,26	0,06
TPA	198-1-1	9,1±0,3	0,54	1,56
	203-7	5,9±0,5	0,35	1,92
CMA	198-1-1	5,5±0,5	0,33	0,31
	203-7	6,2±0,3	0,37	0,35
WA	198-1-1	2,9±0,5	0,17	0,04
	203-7	2,5±0,6	0,15	0,04
V-8	198-1-1	6,2±0,4	0,37	1,44
	203-7	5,9±0,2	0,35	1,42
HA	198-1-1	7,7±0,4	0,46	1,62
	203-7	6,6±0,3	0,39	1,24

Kr – radial growth rate; GC – growth coefficient; PA– potato agar; PACA – potato agar with citric acid; PCA – potato carrot agar; OAT – oatmeal agar; PAVE – potato agar with valerian root extract; TPA – tomato pulp agar; CMA – corn meal agar; WA – water agar; V-8 – vegetable juice agar; HA – hay infusion agar.

Supplementary Figure S2. Growth parameters of *Alternaria* sp. isolates on different nutrient media.

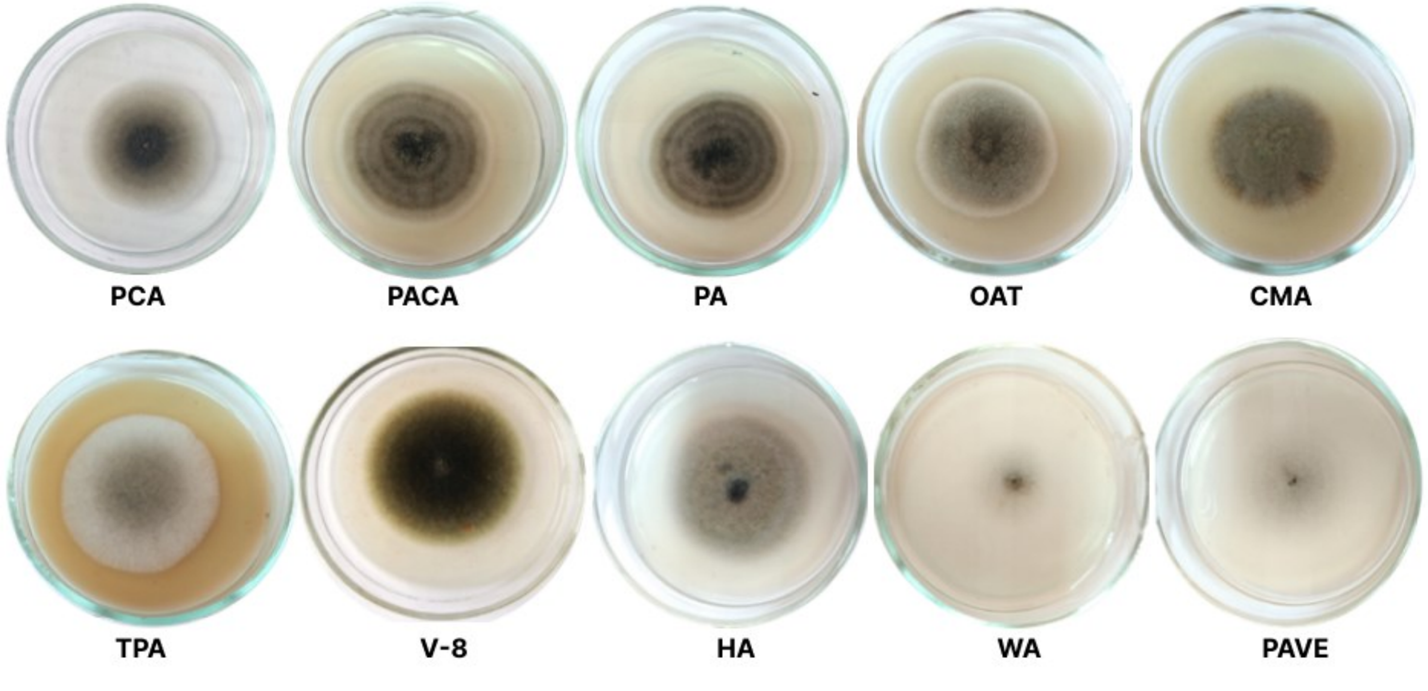

Supplementary Figure S3. Colonies of Alternaria sp. isolate A-203-7 on different nutrient media (7 dpi). PCA – potato carrot agar; PACA – potato agar with citric acid; PA – potato agar; OAT – oatmeal agar; CMA – corn meal agar; TPA – tomato pulp agar; V-8 – vegetable juice agar; HA – hay infusion agar; WA – water agar; PAVE – potato agar with valerian root extract;

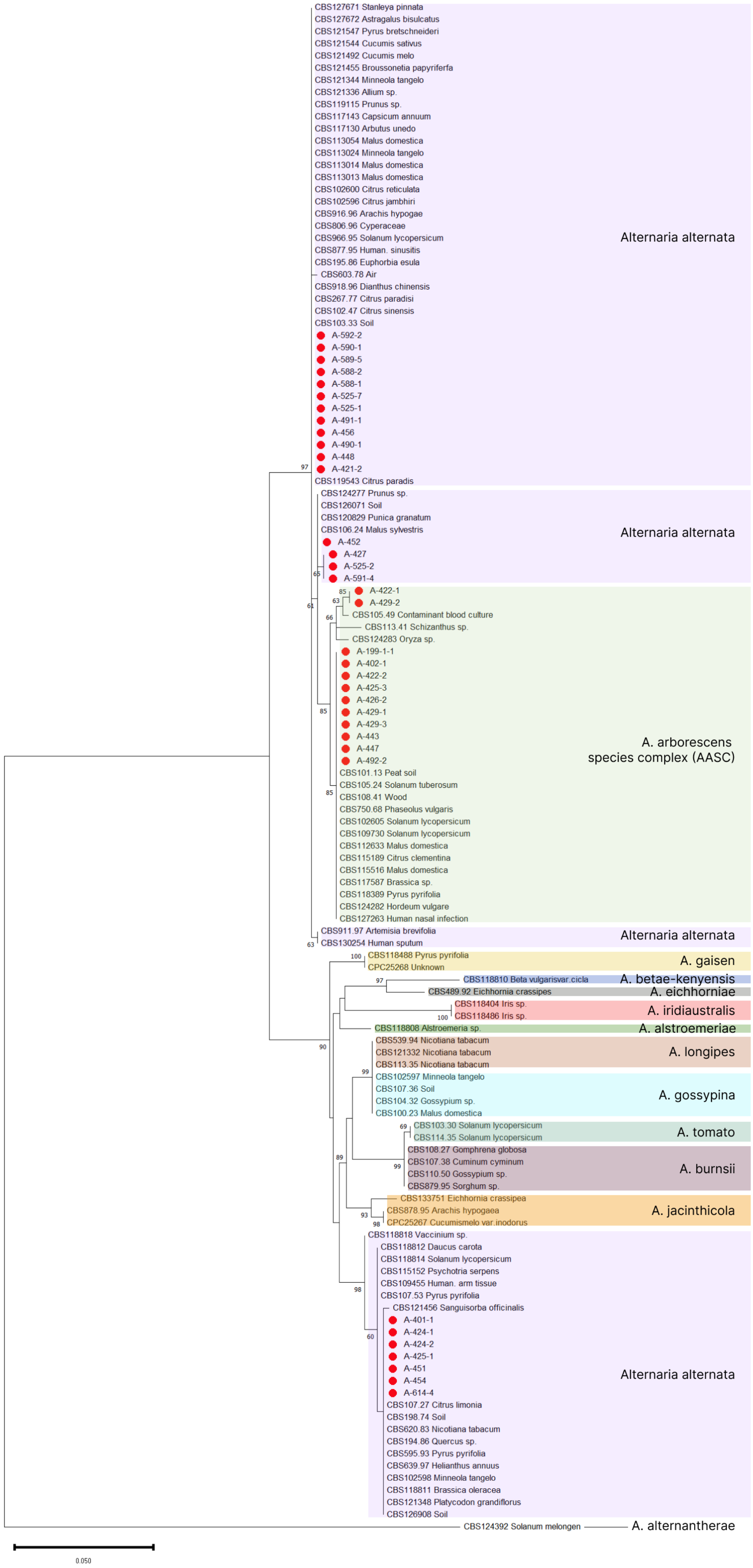

Supplementary Figure S4. Phylogenetic tree constructed by the maximum likelihood method (1,000 bootstrap replicates) using *Alt-a1* marker sequences of *Alternaria* sp. isolates from grapevines obtained in this study (red dot) and representative isolates of the *Alternaria* section. The tree was rooted with *A. alternantherae* (CBS 124392). Only bootstrap values greater than 60 are shown.

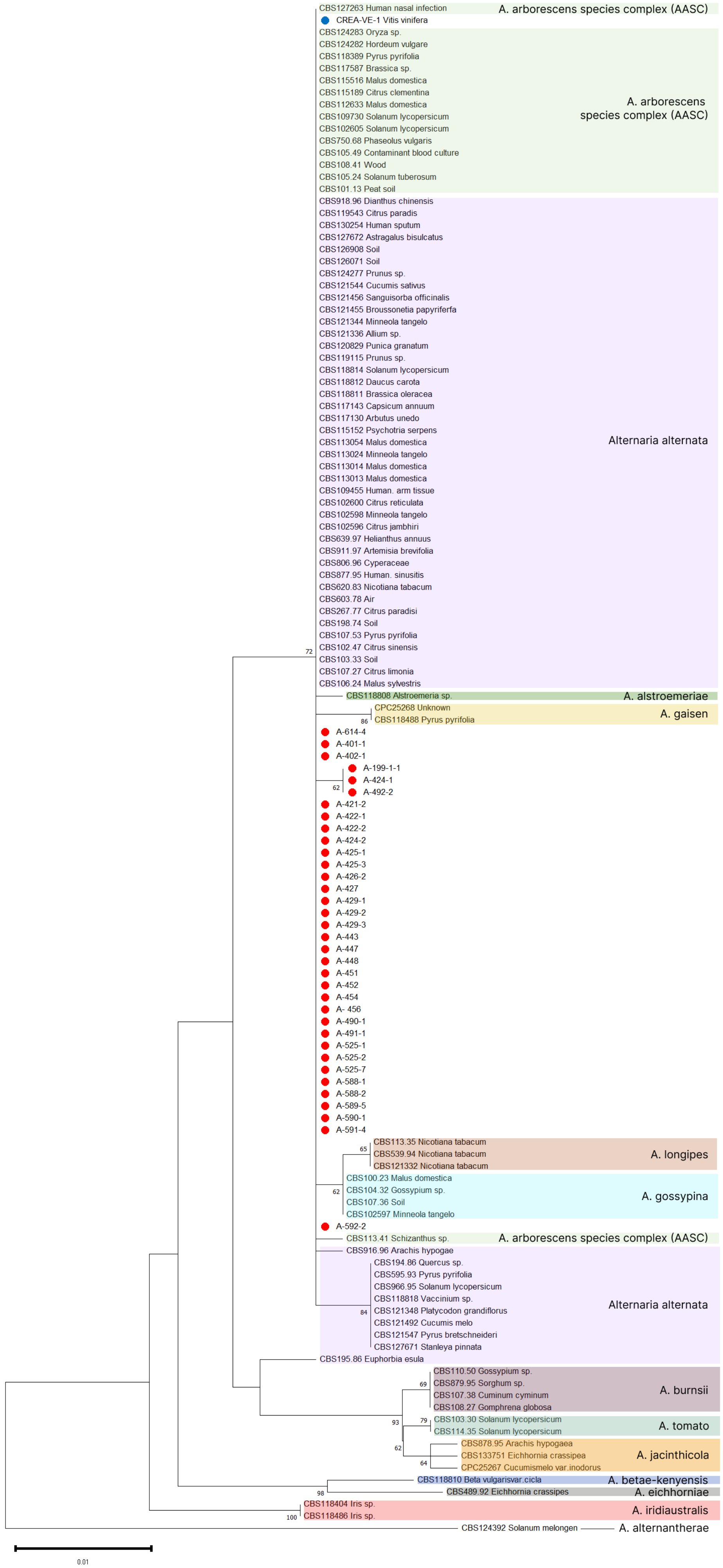

Supplementary Figure S5. Phylogenetic tree constructed by the maximum likelihood method (1,000 bootstrap replicates) using *gapdh* marker sequences of *Alternaria* sp. isolates from grapevines obtained in this study (red dot), isolate from grapevine (Italy) (blue dot) and representative isolates of the *Alternaria* section. The tree was rooted with *A. alternantherae* (CBS 124392). Only bootstrap values greater than 60 are shown.

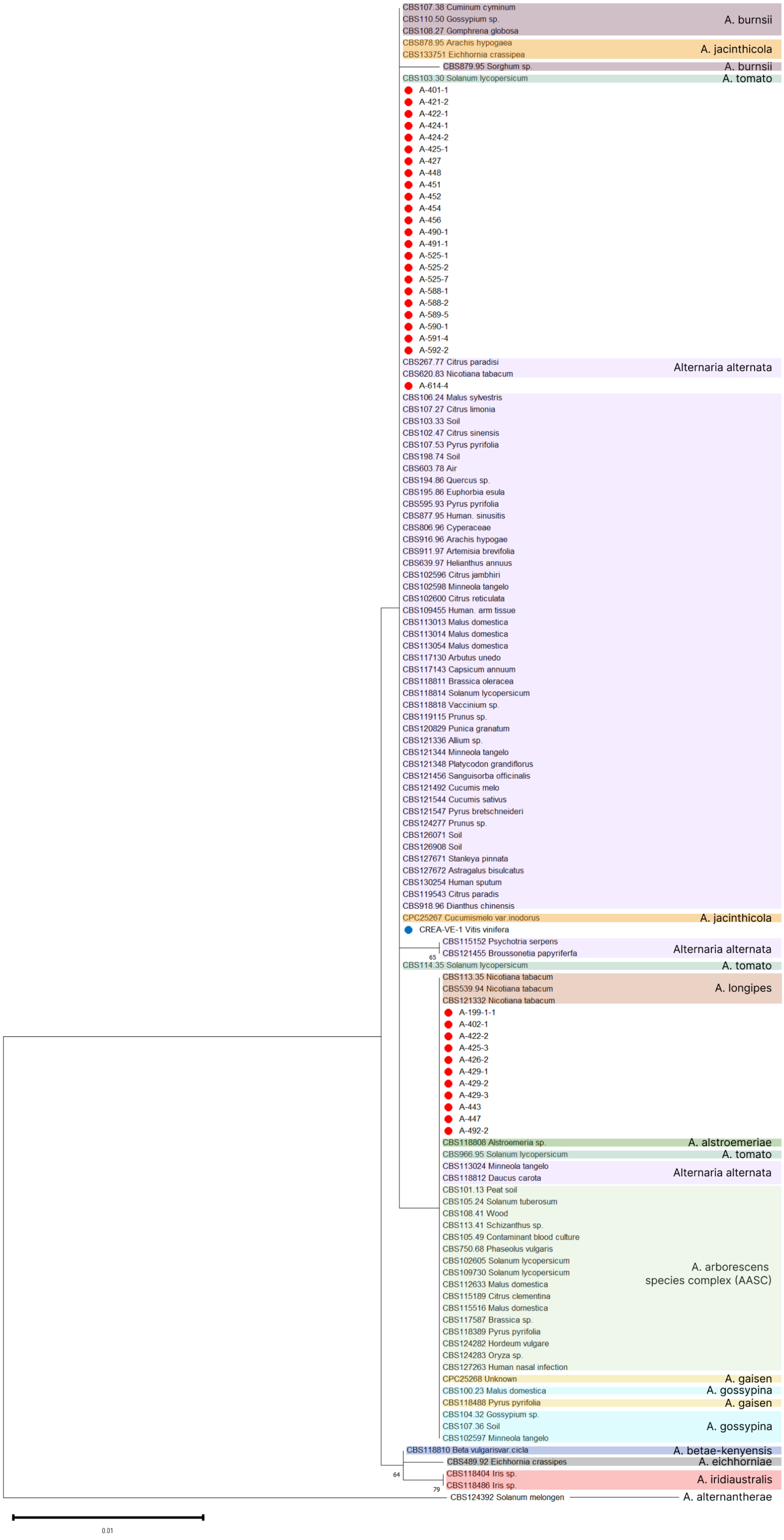

Supplementary Figure S6. Phylogenetic tree constructed by the maximum likelihood method (1,000 bootstrap replicates) using ITS marker sequences of *Alternaria* sp. isolates from grapevines obtained in this study (red dot), isolate from grapevine (Italy) (blue dot) and representative isolates of the *Alternaria* section. The tree was rooted with *A. alternantherae* (CBS 124392). Only bootstrap values greater than 60 are shown.

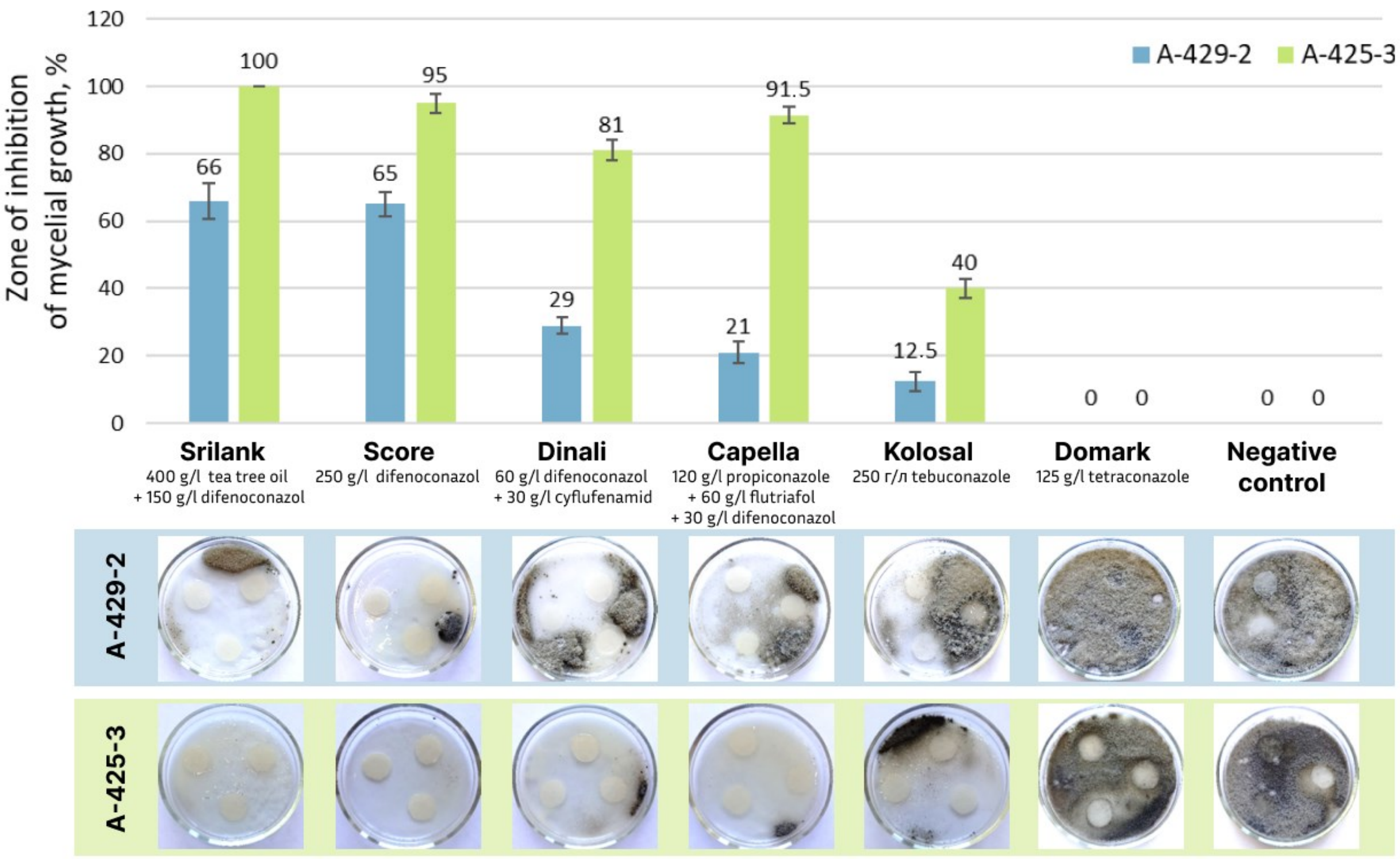

Supplementary Figure S7. Fungicidal activity of chemical preparations substances from the group of triazoles against the *Alternaria* sp. isolates A-425-3 and A-429-2.

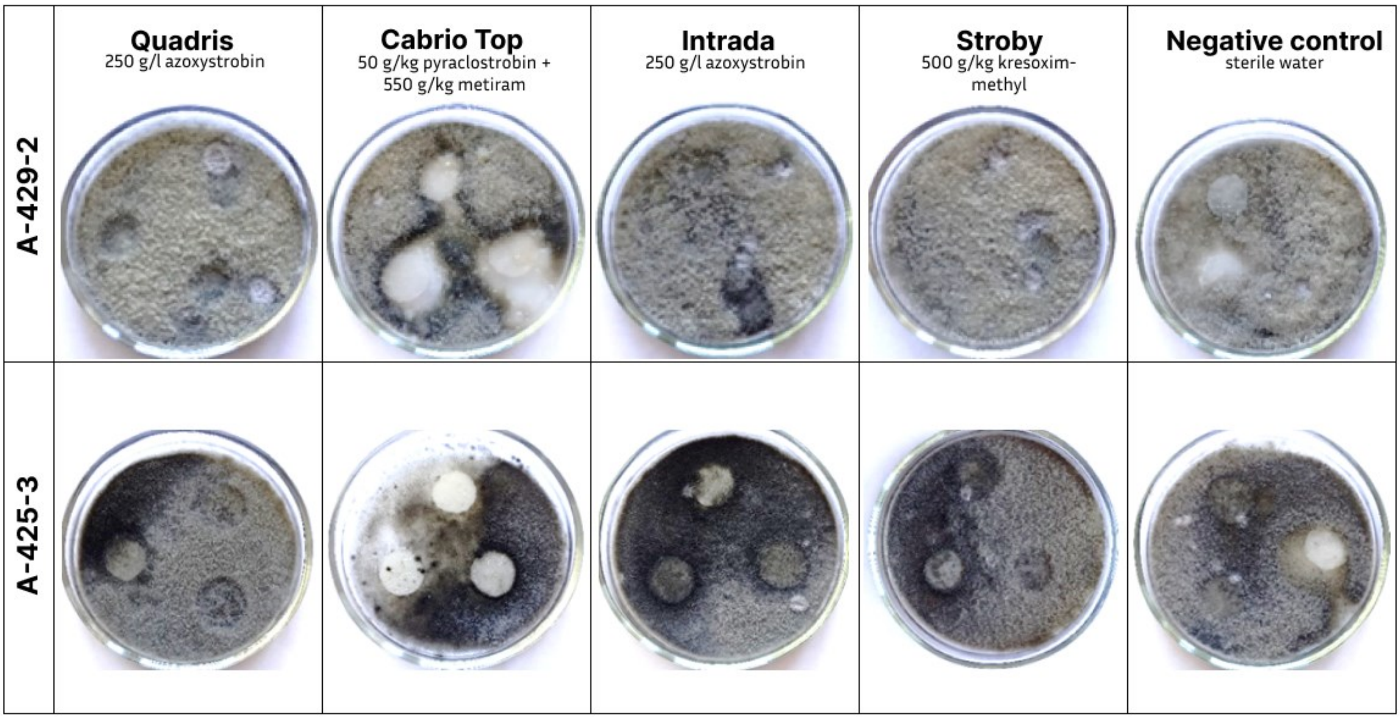

Supplementary Figure S8. Fungicidal activity of chemical preparations substances from the group of strobilurins against the *Alternaria* sp. isolates A-425-3 and A-429-2.

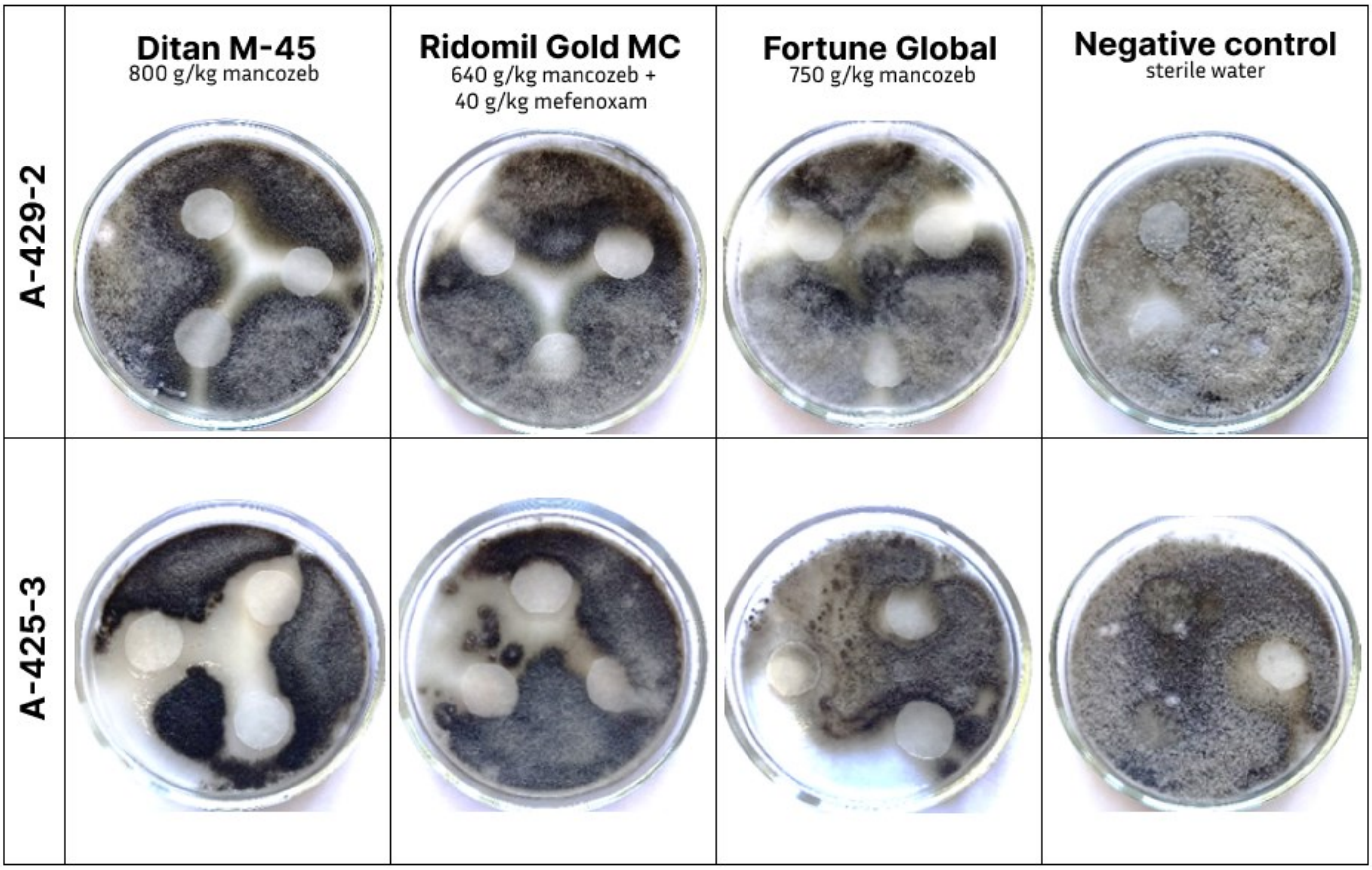

Supplementary Figure S9. Fungicidal activity of chemical preparations substances from the group of dithiocarbamates against the *Alternaria* sp. isolates A-425-3 and A-429-2.

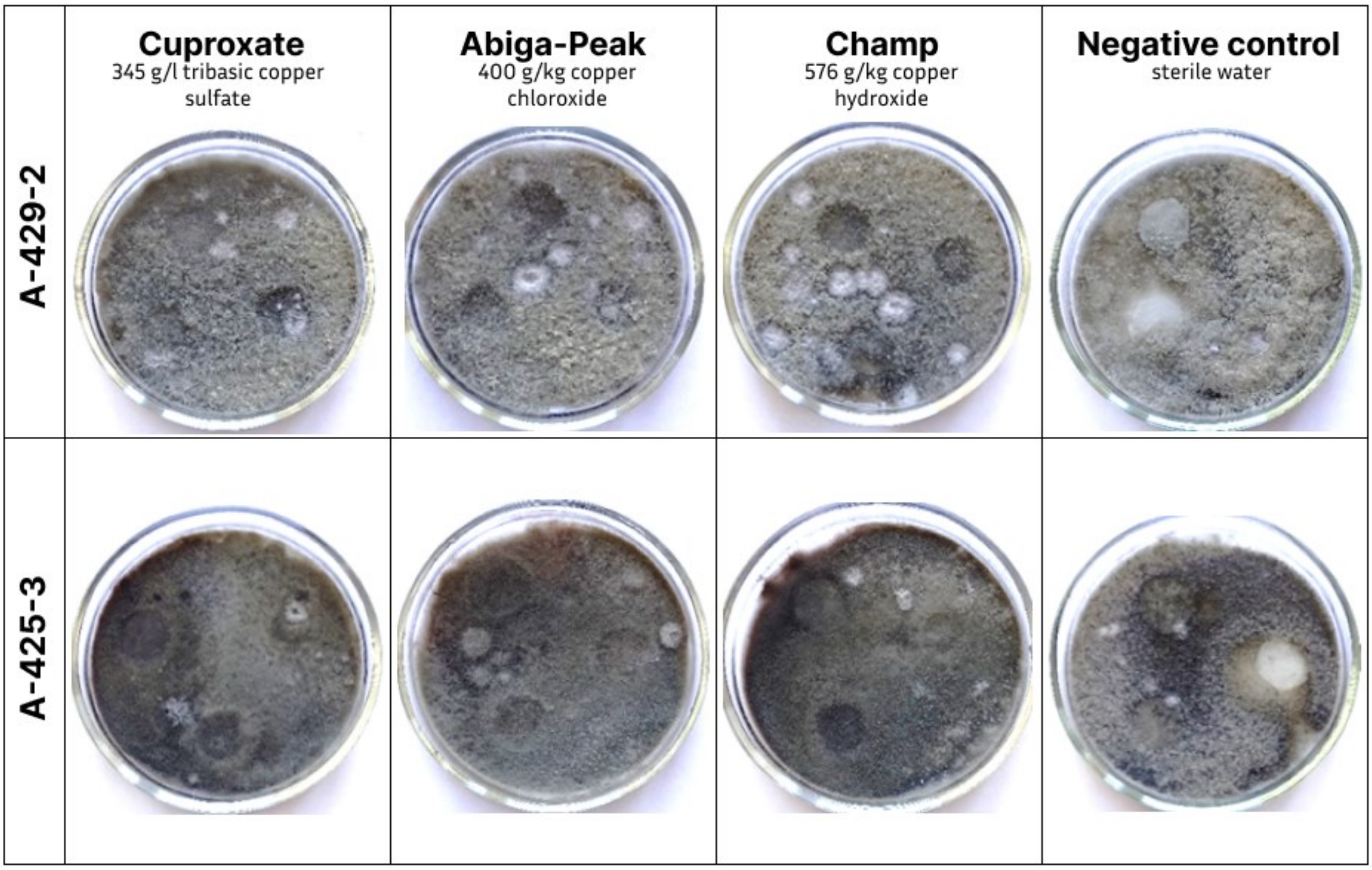

Supplementary Figure S10. Fungicidal activity of copper-based chemicals against strains of *Alternaria* sp. isolated A-425-3 and A-429-2.

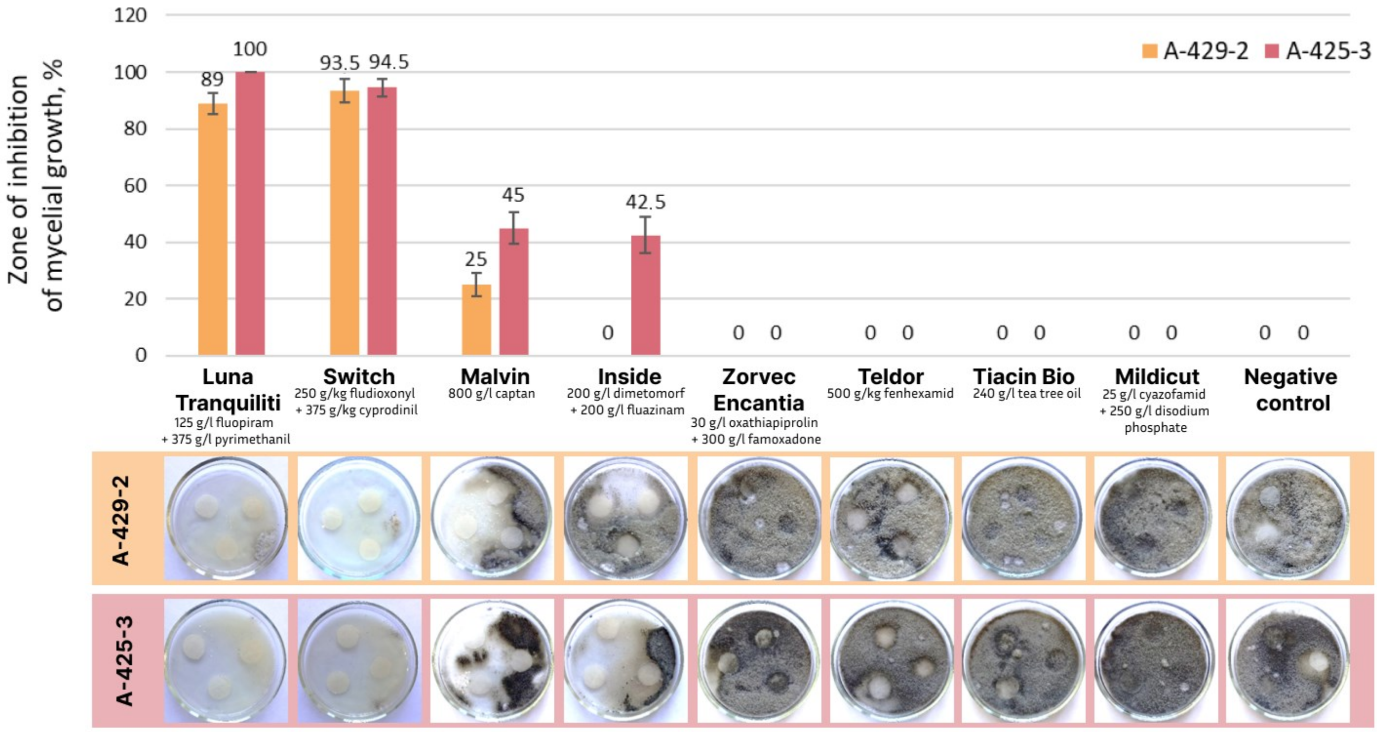

Supplementary Figure S11. Fungicidal activity of chemicals from other chemical groups against isolates *Alternaria* sp. A-425-3 and A-429-2.

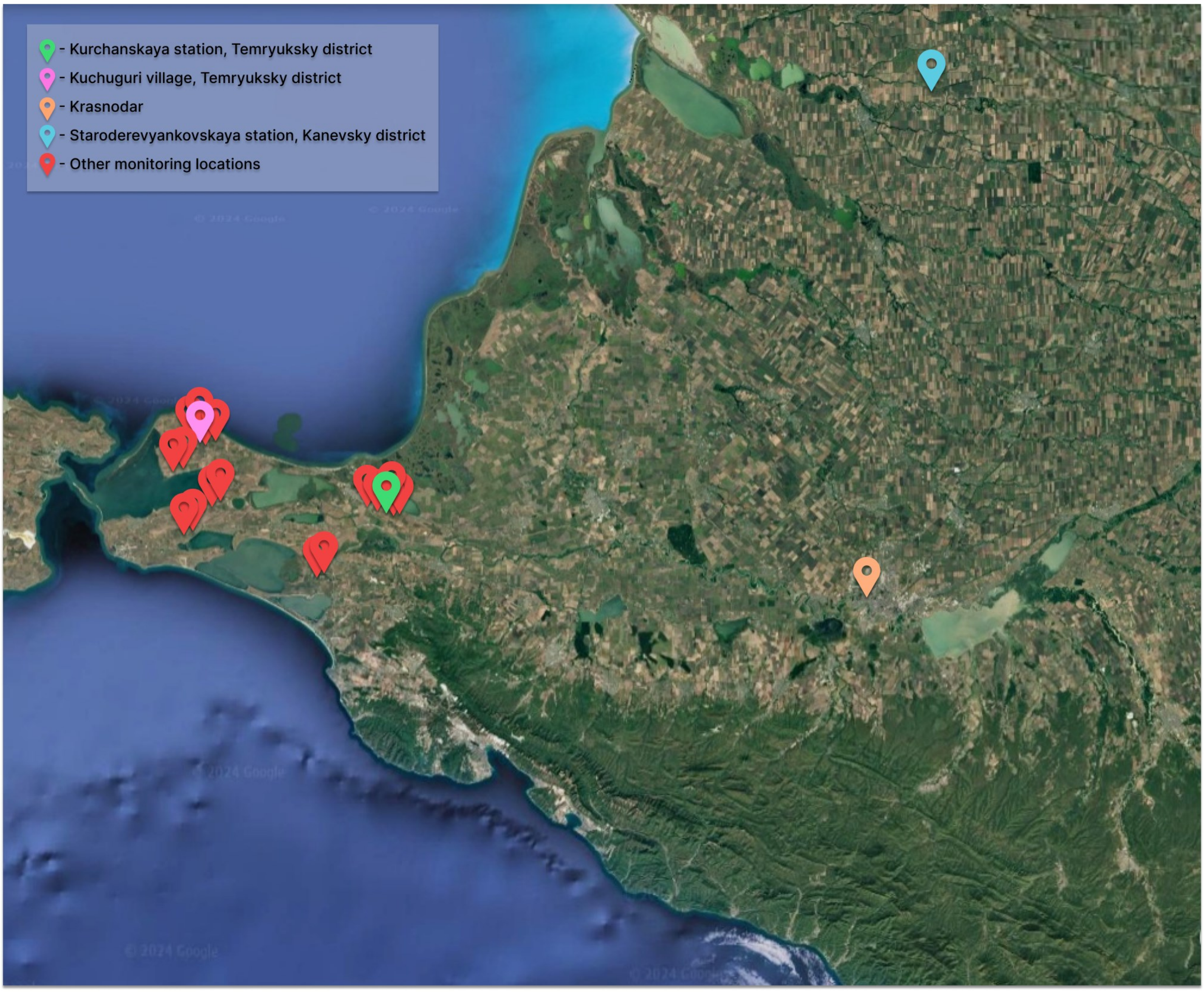

Supplementary Figure S12. Map of the Krasnodar Territory of Russia, where phytosanitary monitoring was carried out.
